# A novel gravity-driven nanofibrous membrane for point-of-use water disinfection: polydopamine-induced *in situ* silver incorporation

**DOI:** 10.1038/s41598-017-02452-2

**Published:** 2017-05-24

**Authors:** Jianqiang Wang, Yichao Wu, Zhe Yang, Hao Guo, Bin Cao, Chuyang Y. Tang

**Affiliations:** 1 Department of Civil Engineering, The University of Hong Kong, Hong Kong, 999077 P. R. China; 2 School of Civil and Environmental Engineering, Nanyang Technological University, 50 Nanyang, Avenue, 639798 Singapore; 3 Singapore Centre for Environmental Life Sciences Engineering, Nanyang Technological University, 60 Nanyang, Avenue, 637551 Singapore

## Abstract

We report a facile method for preparing silver-loaded membranes for point-of-use disinfection and disaster relief applications. A bio-inspired material, polydopamine, was coated onto a highly porous nanofibrous polyacrylonitrile substrate. We then take advantage of the redox properties of polydopamine to form silver nanoparticles *in situ*. These nanoparticles were uniformly distributed on the surface of nanofibers with no apparent agglomeration at a silver loading up to 4.36 wt.% (cPAN-Ag1.5). The silver-incorporated membrane cPAN-Ag1.5 achieved a high pure water flux of 130 Lm^−2^ h^−1^ at 10-cm water head, demonstrating the feasibility of energy-efficient gravity-driven filtration and eliminating the need for electrical power. The strong anti-bacterial activity and high physical rejection of the membrane led to an excellent disinfection power, with no viable bacterial cells detected in its permeate water. The membrane exhibited >7 log reduction for *E. coli* and >6 log reduction for *B. subtilis*. The strategy reported here provides an efficient and green route to synthesize point-of-use membranes. Combining their excellent permeability and disinfection effectiveness, these membranes offer an ideal solution to water supply in disaster-affected areas.

## Introduction

Natural disasters cause loss of life and severe damages of infrastructures. Providing safe drinking water in disaster-affected areas plays a critical role to prevent the outbreak of waterborne diseases in the aftermath. Conventional surface water treatment typically involves coagulation, filtration, and/or chemical disinfection^1, 2^. Alternatively, pressure-driven membrane filtrations such as microfiltration (MF) and ultrafiltration (UF) may be applied for the removal of suspended solids and bacteria. Nevertheless, damaged infrastructure and lacking of reliable power supply often render these conventional water treatment technologies not feasible in disaster-affected areas^3^. An ideal water treatment technology for disaster relieve shall be highly mobile and do not rely on electricity supply to allow its rapid deployment. In addition, high reliability (e.g., disinfection power) and high output (productivity) are required. Whenever possible, on-site chemical dosage should be avoided to ease the logistic requirements and to prevent secondary contamination in disaster-affected areas.

Some examples of point of use technologies for disaster relieve include thermal based processes, media filtration, MF/UF, and forward osmosis (FO)^4–7^. Unfortunately, many of these methods suffer from their relatively low productivity (e.g., thermal processes and FO) or limited treatment efficiency (e.g., media filtration). In recent years, silver-based disinfection has been increasingly used as a novel point-of-use technology for disaster relief because of their powerful biocidal ability^8, 9^. Some notable examples include silver incorporated hydrogels^10^, cryogels^11^, blotting paper^12^, magnetic nanoparticles^13^, silica nanoparticles^14^ and resin beads^15^. More recently, Smith and co-workers^16, 17^ developed silver incorporated ceramic filters for the point-of-use water treatment. Silver nanoparticles incorporated UF, nanofiltration, FO, and hollow fiber membranes were also developed^18–20^. These silver-loaded membranes offer simultaneous physical removal and chemical disinfection. However, the maximum loading is generally limited due to the agglomeration of silver nanoparticles. In addition, the conventional method of loading silver nanoparticles into the membrane matrix reduces the availability of silver nanoparticles due to the limited pore surface area, which will significantly affect their antibacterial effectiveness. Therefore, facile and novel method for surface immobilization of silver nanoparticles needs to be developed.

Polydopamine (PDA) is bioinspired polymer whose molecular structure is similar to that of the adhesive proteins of mussels^21^. PDA can stick onto nearly all kinds of organic and inorganic surfaces through both covalent and noncovalent bonds with the substratum^22, 23^. Membranes coated with PDA have been reported to exhibit an improved antifouling performance due to the presence of catechol and amine groups in PDA^24, 25^. In addition, electrons can be released when catechol groups are oxidized to catecholquinone, and metal ions (e.g., gold and silver) can be reduced at the same time^26^. The bifunctional performance of PDA provides us a simple and green route for the surface immobilization of silver nanoparticles.

In the current study, we aim to fabricate a novel, gravity-driven, water disinfection membrane filter with high water permeability and high disinfection power. Silver nanoparticles are immobilized on the surface of a PDA-coated nanofibrous membrane substrate. The resulting membranes were tested under gravity-driven conditions, and their disinfection properties were systematically investigated. The strategy reported in this study provides a new dimension of designing high-efficiency and high-reliability point-of-use water treatment for disaster relief.

## Results and Discussion

**Figure 1:**
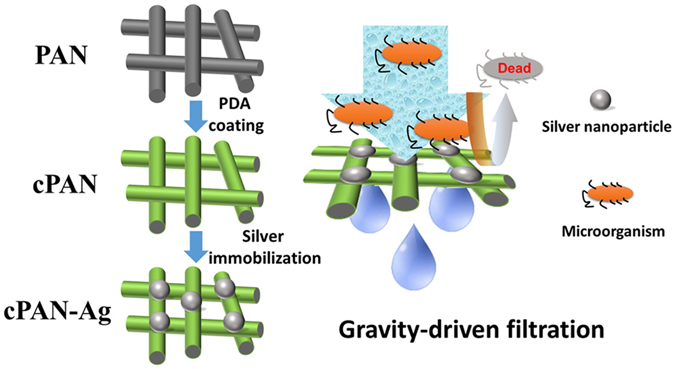
Schematic diagram of preparation and application of the silver-loaded nanofibrous membrane.

**Figure 2:**
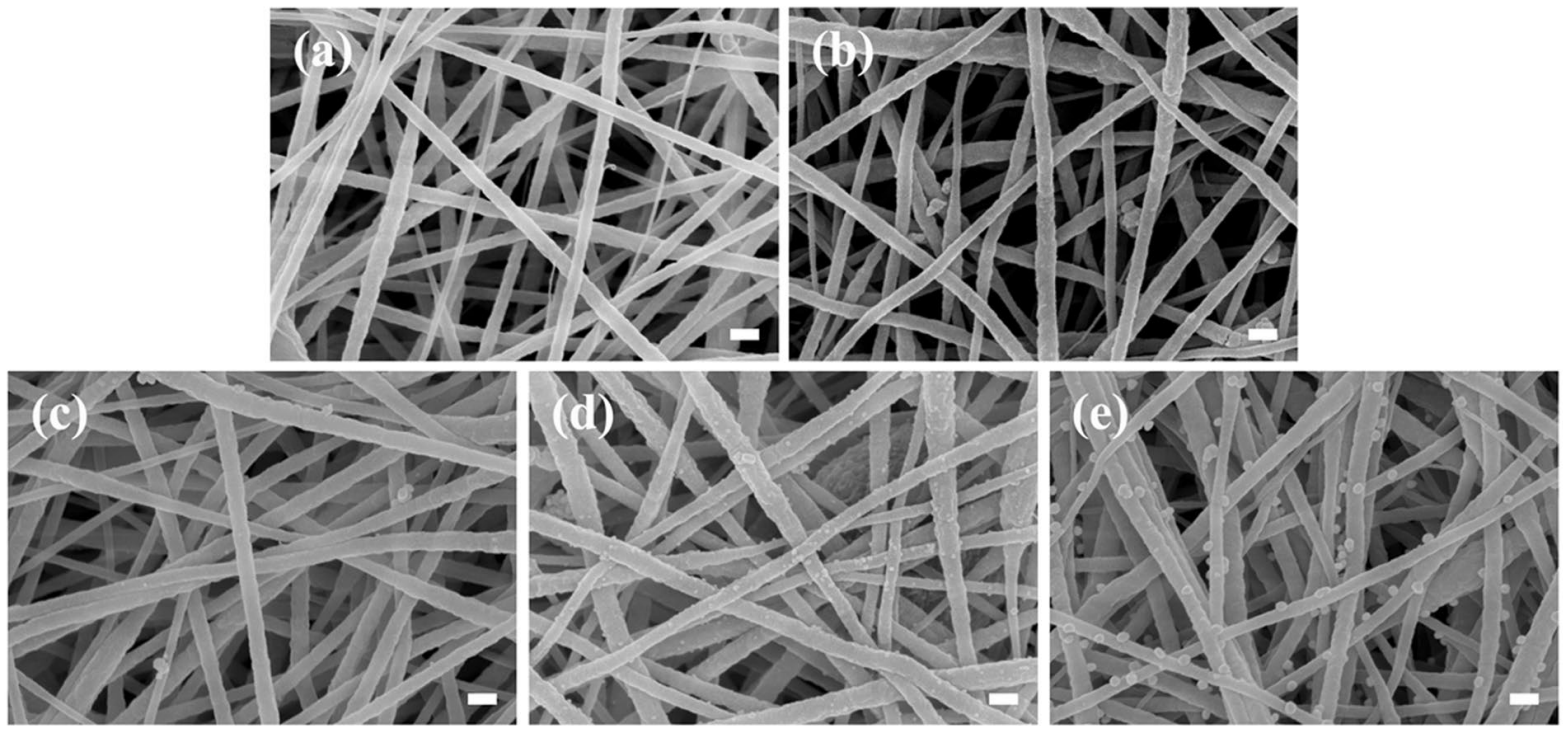
SEM micrographs of PAN (**a**), cPAN (**b**), cPAN-Ag0.5 (**c**), cPAN-Ag1.0 (**d**), and cPAN-Ag1.5 (**e**) nanofibrous membranes (scale bar is 100 nm).

We investigated a novel bio-inspired method for incorporating silver nanoparticles onto a PDA-coated PAN nanofibrous membrane (Fig. 1). Figure 2 shows the SEM micrographs of the pristine PAN, PDA-coated PAN (cPAN), and sliver incorporated cPAN membranes (cPAN-Ag0.5, cPAN-Ag1.0, and cPAN-Ag1.5). The corresponding EDX results are presented in Table 1. The silver loading increased from 0.23 to 4.36 wt.% when the cPAN was exposed to increasing AgNO_3_ concentration from 0.5 to 1.5 g/L. The silver immobilization can be attributed to the catechol groups in PDA^22^. The PDA layer supports an active and green platform for the *in situ* immobilization of silver nanoparticles by its catechol groups, which eliminates the need for reducing agents such as NaBH_4_^11, 14, 27^.

**Table 1:** Physical and chemical properties of the nanofibrous membranes.

	Ag (wt.%)	Porosity	Mean pore size (µm)	Contact angle (°)
PAN	Not detected	89.6%	0.19	<5
cPAN	Not detected	88.9%	0.15	<5
cPAN-Ag0.5	0.23	88.7%	0.14	<5
cPAN-Ag1.0	0.71	87.8%	0.13	<5
cPAN-Ag1.5	4.36	87.1%	0.14	<5

SEM micrographs (Fig. 2) clearly show the immobilized silver nanoparticles for cPAN-Ag1.0 and cPAN-Ag1.5. These nanoparticles were nearly monodispersed and were uniformly distributed on the nanofiber surface. The average size of immobilized particles were 32 ± 6 nm for cPAN-Ag1.0 and 66 ± 8 nm for cPAN-Ag1.5 (Supplementary Fig. S1). In contrast to the conventional method of blending silver nanoparticles into a membrane matrix, we did not observe the commonly encountered agglomeration problem^19, 28^ even at the highest silver loading of 4.36%. Thus, the bio-inspired *in situ* reduction method offers an easy and flexible alternative to prepare a wide range of silver loading with high uniformity and tunable particle size.

Based on the SEM micrographs (Fig. 2), all the membranes appeared to be highly porous with interconnected pore structure. In addition, all the membranes had porosity of greater than 87% (Table 1). Porosity was reduced upon coating of PDA and immobilization of silver nanoparticles. Correspondingly, the effective membrane pore size was reduced from 190 nm for the pristine PAN membrane to 130–140 nm for the silver-loaded membranes (pore size distribution can be found in Supplementary Fig. S2). This observation is also consistent with the SEM micrographs, where the silver-loaded membranes appear to be less porous in comparison with the pristine PAN membrane. The PDA coating together with the *in situ* growth of silver nanoparticles may have taken up some pore space, resulting in a moderate reduction of membrane pore size and porosity.

Contact angle results are shown in Table 1. All the nanofibrous membranes exhibited instant wetting (contact angle <5°) regardless of PDA coating and silver loading. For comparison purpose, an UF membrane prepared by phase inversion showed a contact angle of 41.3 ± 1.4° (Supplementary Fig. S3). Therefore, the super-wetting performance of the nanofibrous membranes can be attributed to the capillary effect for the highly interconnected nanofibrous pore structure.

**Figure 3:**
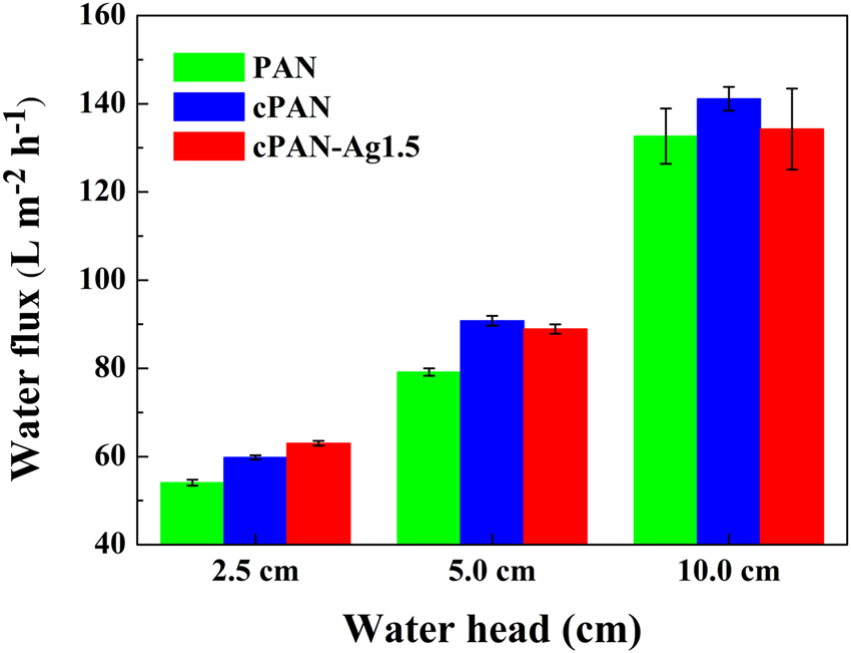
Pure water fluxes of different nanofibrous membranes.

Pure water fluxes of the membranes were tested under gravity driven conditions with water head ranging from 2.5 to 10 cm (Fig. 3). At a water head of merely 10.0 cm, the water flux was as high as 130 L m^−2^ h^−1^ for the PAN membrane. Despite that the silver-loaded cPAN-Ag1.5 membrane had lower porosity and reduced pore diameter (Table 1), its water flux was similar to or greater than the corresponding value of PAN membrane. This result may be partially attributed to the hydrophilic nature of the PDA^24, 29^ as well as the silver nanoparticles^30^. The water permeability of the nanofibrous membranes was approximately 13,000 L m^−2^ h^−1^ bar^−1^, which was much higher than those of conventional MF membranes^31^, thanks to their highly porous structure. The gravity-driven operation of the nanofibrous membranes is advantageous, allowing them to be deployed for point-of-use water filtration without the need for electricity supply.

The rejection of the nanofibrous membrane was determined using silica nanoparticles (diameter of approximately 450 nm) as a surrogate to evaluate the physical removal of water-borne pathogens. In this study, particle rejection was consistently greater than 94%, implying that the nanofibrous membranes were able to achieve a high degree of physical removal as a result of their relatively small membrane pore size (130–190 nm, see Table 1).

**Figure 4:**
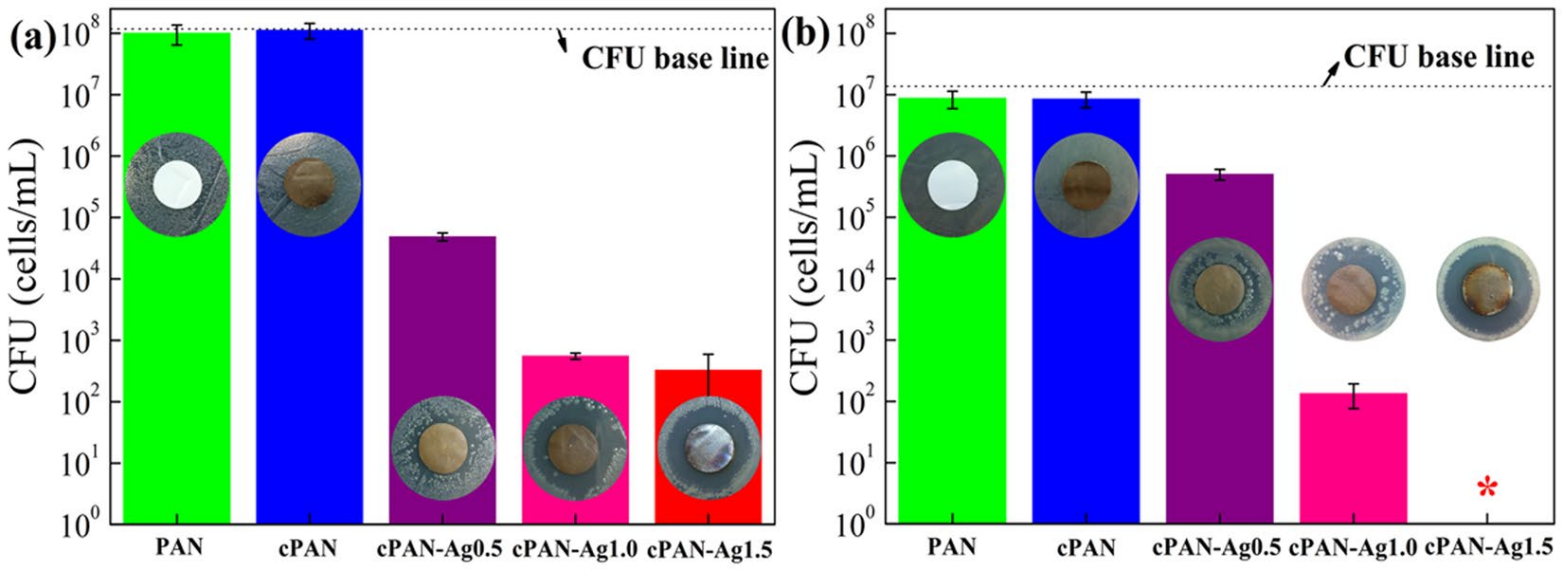
Antibacterial tests (colony forming units and diffusion inhibition zones) for different membranes. Gram-negative *E. coli* (**a**) and Gram-positive *B. subtilis* (**b**) were used as the model bacteria. The symbol “*” stands for below detection limit.

Previous studies have reported that membranes incorporated with silver nanoparticles are capable of inactivating different types of bacteria effectively^19, 30, 32^. The bactericidal effect is mainly caused by release of silver ions, damages to cell membrane via direct contact and generation of reactive oxygen species^15, 33^. To evaluate the disinfection effectiveness of the nanofibrous membranes, membrane antibacterial tests were performed by using *E. coli* and *B. subtilis* as model bacteria (Fig. 4). CFU results showed that the control silver-free membranes PAN and cPAN had no bactericidal effect. In comparison, CFU counting significantly reduced after exposure to cPAN-Ag nanofibrous membrane, and the decrease was more significant at greater silver loading. Membrane cPAN-Ag1.5, with the highest silver loading, exhibited the strongest bactericidal activities (nearly 6 log-reductions for *E. coli* and >7 log-reductions for *B. subtilis*). DIZ also showed similar results (inserts of Fig. 4). DIZs were clearly visible for the silver-incorporated membranes and they became more extended at greater silver loading, with a DIZ of approximately 5 mm obtained for cPAN-Ag1.5.

**Figure 5:**
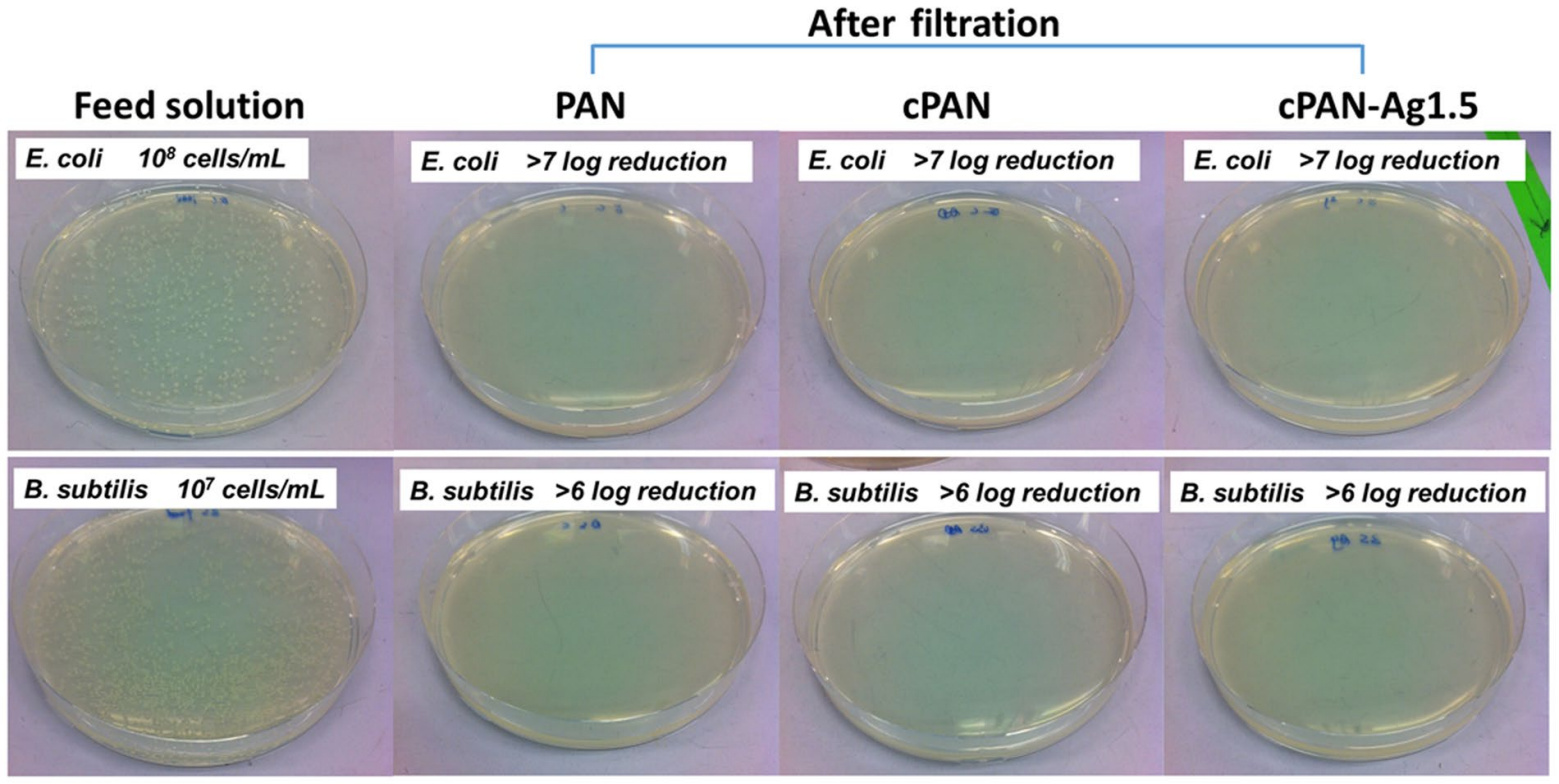
Photographs of colonies formed by *B. subtilis* and *E. coli* in water samples before and after filtration through PAN, cPAN, and cPAN-Ag1.5 nanofibrous membranes.

A simple gravity-driven filtration experiment was designed to test the disinfection performance of the membranes (Fig. 5). No bacteria were detected in permeates, regardless of the membrane used (PAN, cPAN, or cPAN-Ag1.5). Considering the detect limit (~10 cells/mL), membrane filtration achieved at least 7-log and 6-log reductions for *E. coli* and *B. subtilis*, respectively. Since PAN and cPAN had no silver loading and no bactericidal effect (Fig. 4), their disinfection can be attributed to the physical removal noting that their small mean pore size (<200 nm, Table 1) in comparison to the typical size of *E. coli* and *B. subtilis* (1–3 μm^34^). This observation is consistent with the high removal (>94%) obtained for the 450-nm silica particles. Compared to PAN and cPAN, the AgNPs-loaded cPAN-Ag1.5 provides an additional safety barrier due to silver disinfection (see results in Fig. 4). Such additional protection is important in drinking water supply to safeguard against accidental leakages through the membrane.

**Figure 6:**
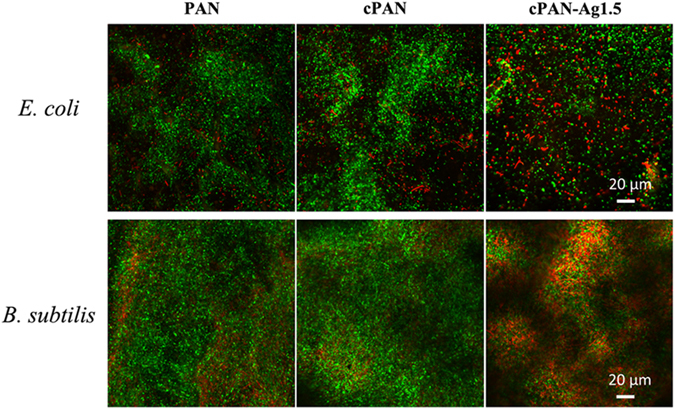
CLSM images of different nanofibrous membranes after filtration the bacteria suspension (green dots and red dots stands for live cells and dead cells respectively).

Figure 6 presents the CLSM micrographs of PAN, cPAN, and cPAN-Ag1.5 after filtering the 10 ml of cell suspension. The live cells are shown in green and dead cells shown in red. The results indicated that the viability ratio (for both *E. coli* and *B. subtilis*) decreased when cPAN-Ag1.5 nanofibrous membranes were used compared to the PAN and cPAN membranes, confirming that bacterial cells were removed and killed by cPAN-Ag1.5 due to the combined physical separation and silver disinfection (detailed live/dead ratio of the cells can be found in Supplementary Fig. S4)^35^. Therefore, cPAN-Ag1.5 membrane can not only remove bacteria from feed solution, but also disinfect bacteria retained on membrane which avoid secondary contamination.

The silver concentration in the permeate water was measured to assess the potential health risks. Our results showed that the leached silver concentration was consistently below 10 μg L^−1^, which is far below the guideline limit of 100 μg L^−1^ for safe drinking water suggested in the World Health Organization^36^.

## Conclusions

The provision of safe drinking water in the aftermath of natural disasters is often hindered by the lack of electricity and infrastructure failures. Point-of-use water disinfection technology can play a vital role in life saving and diseases prevention. Gravity-driven nanofibrous membranes were prepared for point-of-use disinfection, using polydoapmine as a green platform for the *in situ* formation of uniformly-dispersed silver nanoparticles. The amount and size of silver nanoparticles was controlled by the concentration of silver nitrate, with greater loading and particle size observed at higher silver concentration. The silver-loaded membrane cPAN-Ag1.5 showed excellent antibacterial efficacy against *E. coli* and *B. subtilis* in both CFU and DIF tests. In addition, it had a high water flux (130 Lm^−2^ h^−1^ at 10-cm water head) and high particle rejection (>94% removal of 450-nm silica nanoparticles). When applied in gravity-driven filtration experiments, the membrane achieved >7 and >6 log reduction for *E. coli* and *B. subtilis*, respectively, with no viable cells detected in the permeate water. Meanwhile, the leached silver concentration in the permeate water was greatly below the WHO guideline value to allow its use as the safe drinking water. The reported gravity-driven nanofibrous water disinfection membrane has the promising applications in disaster relief and emergency water treatment.

## Methods

### Chemicals

Polyacrylonitrile (PAN, Mw = 150,000), dimethylformamide (DMF), and tris (hydroxymethyl) aminomethane (>99%) were purchased from Sigma-Aldrich. Dopamine hydrochloride (99%) and silver nitrate (AgNO_3_, 99%) were purchased from Alfa Aesar. Silica nanoparticles used for membrane rejection tests were obtained from XFNano (China). The manufacturer reports a mean particle diameter of 500 nm.

### Membrane preparation and modification

PAN nanofibrous membranes were synthesized using the electrospun method^37^. Figure 1 shows the schematics for membrane surface modification. The PAN nanofibrous membrane was immersed in a dopamine Tris-HCl solution (2.0 g/L PDA, 10 mM Tris-HCl, pH 8.5) under continuous shaken for 12 h at 25 °C. The self-polymerization of dopamine led to a PDA coating on the nanofibrous substrate^37^. The PDA-coated PAN membranes (cPAN) were rinsed using deionized water.

Silver nanoparticles were immobilized onto cPAN membranes through a spontaneous reduction of silver ions *in situ* by PDA^32^. Typically, a cPAN coupon (diameter of 7.6 cm) was immersed into a silver nitrate solution (0.5, 1.0, or 1.5 g/L) for 5.0 h. The silver ions were reduced by the catechol groups in the PDA chain. During the duration, the solution was covered by aluminum foil and was shaken continuously. The resulting silver-loaded membranes were thoroughly rinsed with deionized water. They were denoted as cPAN-Ag0.5, cPAN-Ag1.0 and cPAN-Ag1.5, respectively.

### Membrane characterization

The structure, morphology, and chemical composition of the membranes were characterized using a scanning electron microscope (SEM, LEO 1530 FEG, LEO, UK) equipped with an energy-dispersive X-ray spectroscopy (EDX) unit.

Membrane contact angle was measured by a Contact Angle Analyzer (Powereach^®^, China) using the sessile drop method. Pore size was tested using capillary flow porometry (Porolux 1000, Germany) using pressures ranging from 0 to 2.3 bar. Porosity (*ε*) was determined by measuring the dry mass (m_dry_) and wet mass (m_wet_) of membrane samples using equation (1)^38^:

1 $${\rm{\varepsilon }}=\frac{({m}_{wet}-{m}_{dry})/{\rho }_{w}}{(\frac{{m}_{wet}-{m}_{dry}}{{\rho }_{w}})+({m}_{dry}/{\rho }_{p})}\times 100 \% $$

where ρ_w_ and ρ_p_ are density of wetting solvent (deionized water) and polymer, respectively.

Water flux was tested in a dead-end stirred cell (Model 8400, Millipore, USA) under gravity-driven conditions. Briefly, a piece of nanofibrous membrane (effective area of 41.6 cm^2^) was mounted to the cell. Filtration experiments were performed at different water head (2.5, 5.0 and 10.0 cm). Water flux was determined based on the volume of permeate water collected within a given time interval. Rejection for silica particles (which were pre-treated with ultrasonication for 30 min to avoid aggregation) was calculated based on the particle concentration in the permeate water and that the feed water.

Silver leaching tests were performed by filtering deionized water at a water head of 10 cm through the dead-end stirred cell. Samples (5 mL) were taken at predetermined time intervals and their silver concentrations were measured using inductively coupled plasma optical emission spectrometer (ICP-OES, PE Optima 8300, USA).

### Membrane antibacterial tests

Colony formation units and diffusion inhibition zone tests were performed following our existing procedures^19^. Gram-positive *Bacillus subtilis* 168 and Gram-negative *Escherichia coli* K12 were used as model organisms. After overnight growth in LB broth, cells were centrifuged and rinsed twice^39^. The antibacterial effect was assessed by immersing membrane discs (diameter ~12.7 cm) into bacterial suspension on a rotary shaker (150 rpm) at 25 °C. Cell density of *E. coli* and *B. subtilis* was approximately 10^8^/mL and 10^7^/mL, respectively. After about 10–24 h of exposure, bacterial cell viability was quantified by the drop-plate method^40, 41^. Three replicates were performed for each membrane sample.

Diffusion inhibition zone tests were also performed with three replicates for each membrane sample. For each test, 100 µl of bacterial culture was spread on an LB agar plate. A membrane disc (diameter ~12.7 mm) was placed on each agar plate. After 24-hour incubation under optimal temperature condition (37 °C for *E. coli* and 30 °C for *B. subtilis*), the width of diffusion inhibition zone around membrane was measured.

### Membrane filtration and disinfection

A piece of membrane with an effective area of 7.07 mm^2^ was mounted onto the tip of a 25-mL polystyrene pipette (Corning, Inc.). To characterize the disinfection effect, both *E. coli* and *B. subtilis* were used as bacterial indicators for gravity filtration. Typically, a 10.0 mL of bacterial suspension (10^8^ and 10^7^ cells/mL for *E. coli* and *B. subtilis* respectively) was injected into the pipette for filtration with an initial water head of 10.2 cm. Filtration experiments were carried out with three replicates for each membrane for a duration of 4 h. Permeates were collected and spread on LB agar plate to quantify cultivable cells. Viability of the bacterial cells retained on the membranes were determined using the LIVE/DEAD BacLight Bacterial Viability Kit L7012 (Molecular Probes Inc.) and visualized by Carl Zeiss Confocal Laser Scanning Microscopy (CLSM) LSM 780^42^.

## Electronic supplementary material

Revised supplementary information

## References

[1] Clasen TF, Cairncross S (2004). Editorial: Household water management: refining the dominant paradigm. Trop. Med. Int. Health.

[2] Yarlagadda S, Gude VG, Camacho LM, Pinappu S, Deng S (2011). Potable water recovery from As, U, and F contaminated ground waters by direct contact membrane distillation process. J. Hazard. Mater..

[3] Loo S-L, Fane AG, Krantz WB, Lim T-T (2012). Emergency water supply: A review of potential technologies and selection criteria. Water Res.

[4] Clasen TF, Thao DH, Boisson S, Shipin O (2008). Microbiological effectiveness and cost of boiling to disinfect drinking water in rural Vietnam. Environ. Sci. Technol..

[5] Sobsey MD, Stauber CE, Casanova LM, Brown JM, Elliott MA (2008). Point of use household drinking water filtration: A practical, effective solution for providing sustained access to safe drinking water in the developing world. Environ. Sci. Technol..

[6] Peter-Varbanets M, Zurbrugg C, Swartz C, Pronk W (2009). Decentralized systems for potable water and the potential of membrane technology. Water Res.

[7] Butler E (2013). Point of use water treatment with forward osmosis for emergency relief. Desalination.

[8] Li Q (2008). Antimicrobial nanomaterials for water disinfection and microbial control: potential applications and implications. Water Res.

[9] Liu L, Bai H, Liu J, Sun DD (2013). Multifunctional graphene oxide-TiO_2_-Ag nanocomposites for high performance water disinfection and decontamination under solar irradiation. J. Hazard. Mater..

[10] Zeng X, McCarthy DT, Deletic A, Zhang X (2015). Silver/reduced graphene oxide hydrogel as novel bactericidal filter for point-of-use water disinfection. Adv. Funct. Mater..

[11] Loo S-L (2013). Superabsorbent cryogels decorated with silver nanoparticles as a novel water technology for point-of-use disinfection. Environ. Sci. Technol..

[12] Dankovich TA, Gray DG (2011). Bactericidal paper impregnated with silver nanoparticles for point-of-use water treatment. Environ. Sci. Technol..

[13] Liu J, Zhao Z, Feng H, Cui F (2012). One-pot synthesis of Ag–Fe_3_O_4_ nanocomposites in the absence of additional reductant and its potent antibacterial properties. J. Mater. Chem..

[14] He D, Kacopieros M, Ikeda-Ohno A, Waite TD (2014). Optimizing the design and synthesis of supported silver nanoparticles for low cost water disinfection. Environ. Sci. Technol..

[15] Lin S (2013). Silver nanoparticle-alginate composite beads for point-of-use drinking water disinfection. Water Res..

[16] Oyanedel-Craver VA, Smith JA (2008). Sustainable colloidal-silver-impregnated ceramic filter for point-of-use water treatment. Environ. Sci. Technol..

[17] Abebe LS, Su YH, Guerrant RL, Swami NS, Smith JA (2015). Point-of-use removal of cryptosporidium parvum from water: independent effects of disinfection by silver nanoparticles and silver ions and by physical filtration in ceramic porous media. Environ. Sci. Technol..

[18] Park SY, Chung JW, Chae YK, Kwak SY (2013). Amphiphilic thiol functional linker mediated sustainable anti-biofouling ultrafiltration nanocomposite comprising a silver nanoparticles and poly(vinylidene fluoride) membrane. ACS Appl. Mater. Interfaces.

[19] Liu X (2013). Synthesis and characterization of novel antibacterial silver nanocomposite nanofiltration and forward osmosis membranes based on layer-by-layer assembly. Water Res.

[20] Booshehri AY, Wang R, Xu R (2013). The effect of re-generable silver nanoparticles/multi-walled carbon nanotubes coating on the antibacterial performance of hollow fiber membrane. Chem. Eng. J.

[21] Tang L, Livi KJT, Chen KL (2015). Polysulfone membranes modified with bioinspired polydopamine and silver nanoparticles formed *in situ* to mitigate biofouling. Environ. Sci. Technol. Lett..

[22] Lee H, Dellatore SM, Miller WM, Messersmith PB (2007). Mussel-inspired surface chemistry for multifunctional coatings. Science.

[23] Liu XS (2013). Mussel-inspired polydopamine: a biocompatible and ultrastable coating for nanoparticles *in vivo*. ACS Nano.

[24] McCloskey BD (2012). A bioinspired fouling-resistant surface modification for water purification membranes. J. Membr. Sci..

[25] Jiang JH (2013). Antifouling and antimicrobial polymer membranes based on bioinspired polydopamine and strong hydrogen-bonded poly(N-vinyl pyrrolidone). ACS Appl. Mater. Interfaces.

[26] Hong S (2011). Bio-inspired strategy for on-surface synthesis of silver nanoparticles for metal/organic hybrid nanomaterials and LDI-MS substrates. Nanotechnology.

[27] Agnihotri S, Mukherji S, Mukherji S (2013). Immobilized silver nanoparticles enhance contact killing and show highest efficacy: elucidation of the mechanism of bactericidal action of silver. Nanoscale.

[28] Khin MM, Nair AS, Babu VJ, Murugan R, Ramakrishna S (2012). A review on nanomaterials for environmental remediation. Energy Environ. Sci..

[29] Arena JT, Manickam SS, Reimund KK, Freeman BD, McCutcheon JR (2014). Solute and water transport in forward osmosis using polydopamine modified thin film composite membranes. Desalination.

[30] Huang L (2016). *In situ* immobilization of silver nanoparticles for improving permeability, antifouling and anti-bacterial properties of ultrafiltration membrane. J. Membr. Sci..

[31] Scharnagl N, Buschatz H (2001). Polyacrylonitrile (PAN) membranes for ultra- and microfiltration. Desalination.

[32] Yang Z, Wu Y, Wang J, Cao B, Tang CY (2016). *In situ* reduction of silver by polydopamine: A novel antimicrobial modification of a thin-film composite polyamide membrane. Environ. Sci. Technol..

[33] Morones JR (2005). The bactericidal effect of silver nanoparticles. Nanotechnology.

[34] Han Y, Xu Z, Gao C (2013). Ultrathin graphene nanofiltration membrane for water purification. Adv. Funct. Mater..

[35] Tamboli DP, Lee DS (2013). Mechanistic antimicrobial approach of extracellularly synthesized silver nanoparticles against gram positive and gram negative bacteria. J. Hazard. Mater..

[36] Organization, W. H. *Guidelines for drinking-water quality: recommendations*. Vol. 1 (World Health Organization, 2011).

[37] Wang J, Guo H, Yang Z, Mei Y, Tang CY (2017). Gravity-driven catalytic nanofibrous membranes prepared using a green template. J. Membr. Sci..

[38] Wei J, Qiu C, Tang CY, Wang R, Fane AG (2011). Synthesis and characterization of flat-sheet thin film composite forward osmosis membranes. J. Membr. Sci..

[39] Liu X, Jin X, Cao B, Tang CY (2014). Bactericidal activity of silver nanoparticles in environmentally relevant freshwater matrices: Influences of organic matter and chelating agent. J. Environ. Chem. Eng..

[40] Chen C-Y, Nace GW, Irwin PL (2003). A 6 × 6 drop plate method for simultaneous colony counting and MPN enumeration of Campylobacter jejuni, Listeria monocytogenes, and Escherichia coli. J. Microbiol. Methods.

[41] Wu Y, Shukal S, Mukherjee M, Cao B (2015). Involvement in denitrification is beneficial to the biofilm lifestyle of comamonas testosteroni: a mechanistic study and its environmental implications. Environ. Sci. Technol..

[42] Yu Y, Wu Y, Cao B, Gao Y-G, Yan X (2015). Adjustable bidirectional extracellular electron transfer between Comamonas testosteroni biofilms and electrode via distinct electron mediators. Electrochem. Commun..

