# Supplementary material for: A novel gravity-driven nanofibrous membrane for point-of-use water disinfection: polydopamine-induced in situ silver incorporation
Source: Sci Rep. 2017 May 24;7:2334. doi: 10.1038/s41598-017-02452-2 (PMC5443768; doi:10.1038/s41598-017-02452-2)
Supplement: Supplementary file 1 — Revised supplementary information [file 41598_2017_2452_MOESM1_ESM.doc]

Supplementary Information

**A novel gravity-driven nanofibrous membrane for point-of-use water disinfection: polydopamine-induced *in situ* silver incorporation**

Jianqiang Wang ^a^, Yichao Wu^b,c^, Zhe Yang ^a^, Hao Guo ^a^, Bin Cao ^b,c^, and Chuyang Y. Tang ^a^*

^a^ Department of Civil Engineering, The University of Hong Kong, Hong Kong, 999077, P. R. China.

^b^ School of Civil and Environmental Engineering, Nanyang Technological University, 50 Nanyang Avenue, 639798, Singapore.

^c^ Singapore Centre for Environmental Life Sciences Engineering, Nanyang Technological University, 60 Nanyang Avenue, 637551, Singapore.

*corresponding author: tangc@hku.hk


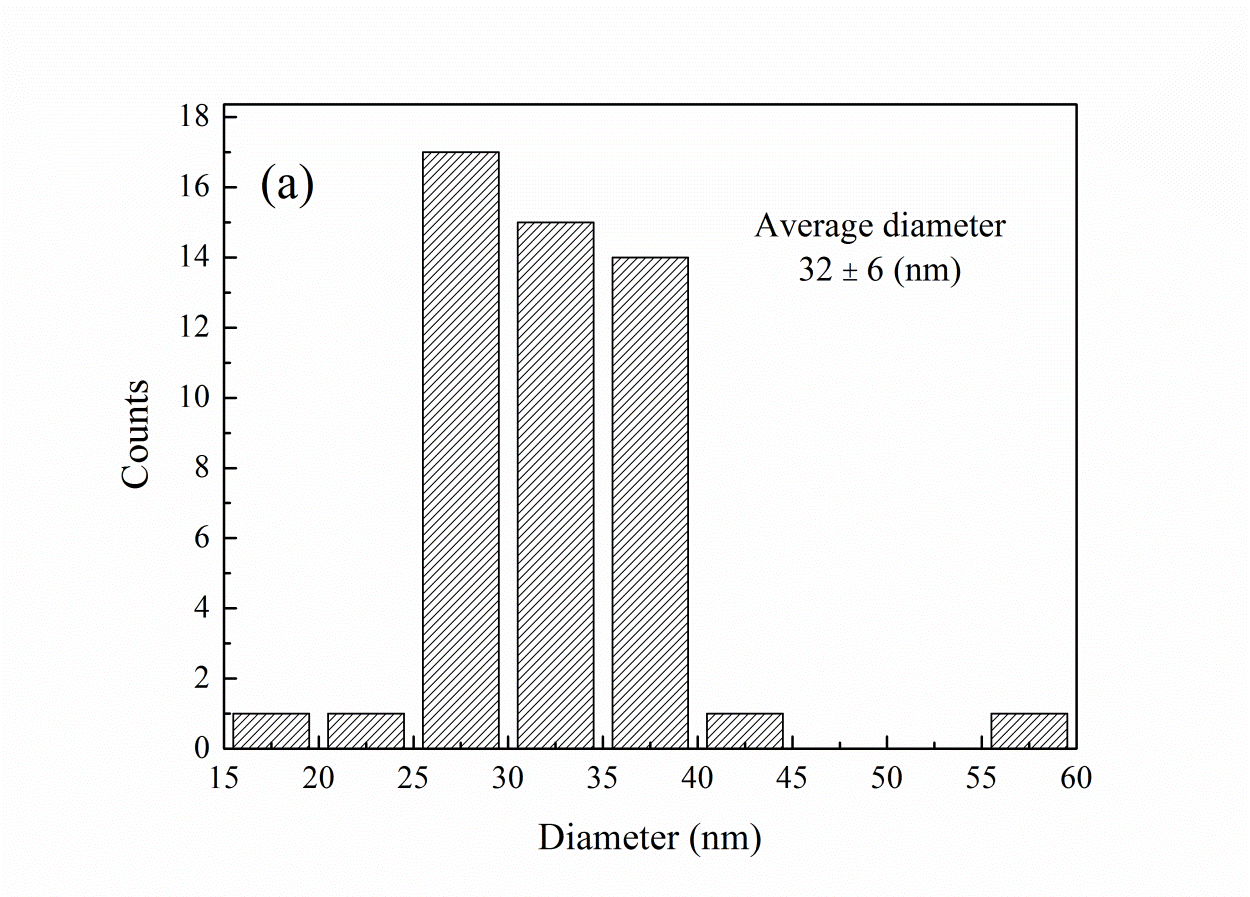


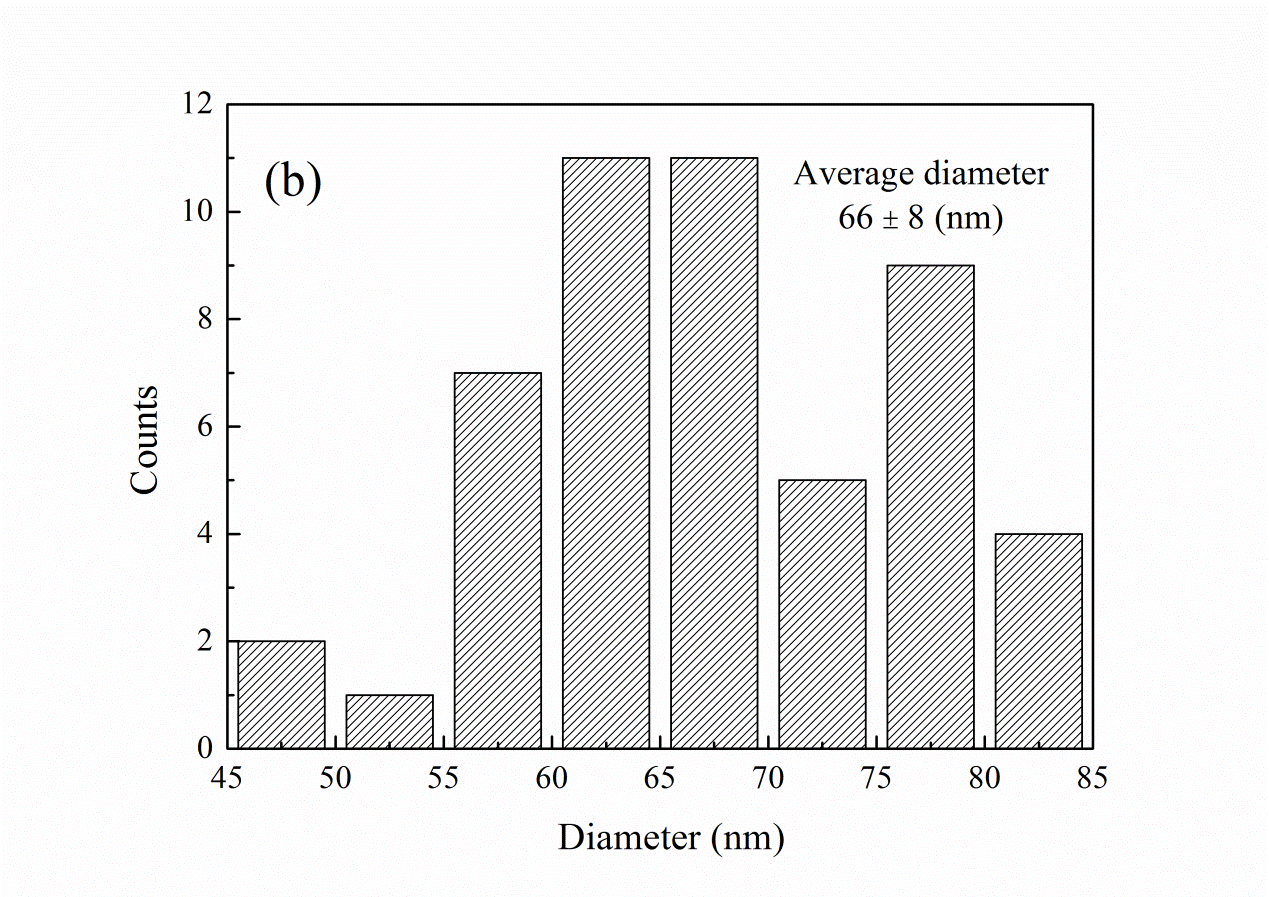


**Fig. S1.** Size distribution of the silver nanoparticles loaded on the cPAN-Ag1.0 (a) and cPAN-Ag1.5 (b) nanofibrous membrane.


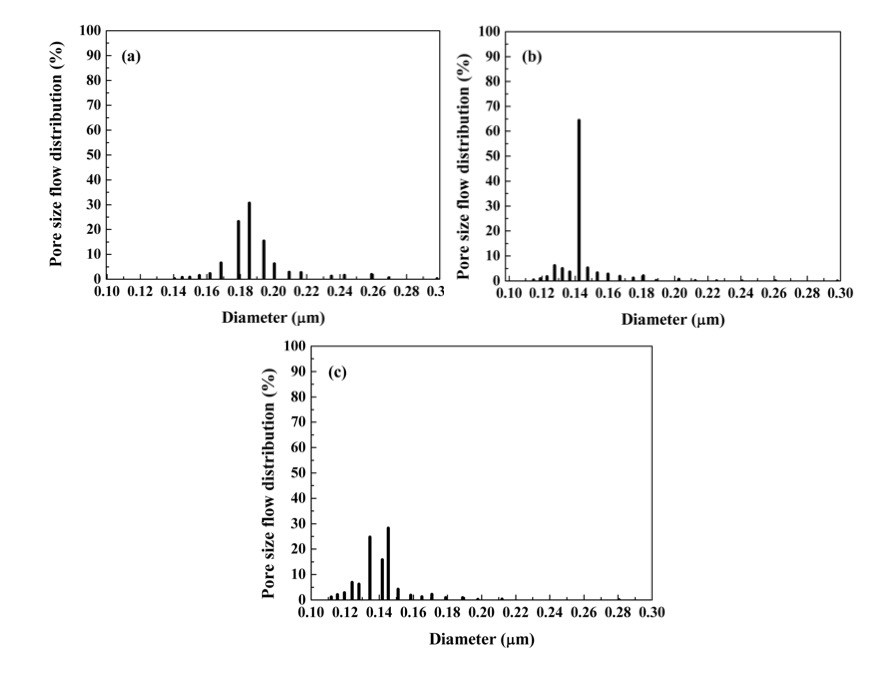


**Fig. S2.** Pore size distribution of the PAN (a), cPAN (b) and cPAN-Ag1.5 (c) nanofibrous membrane.


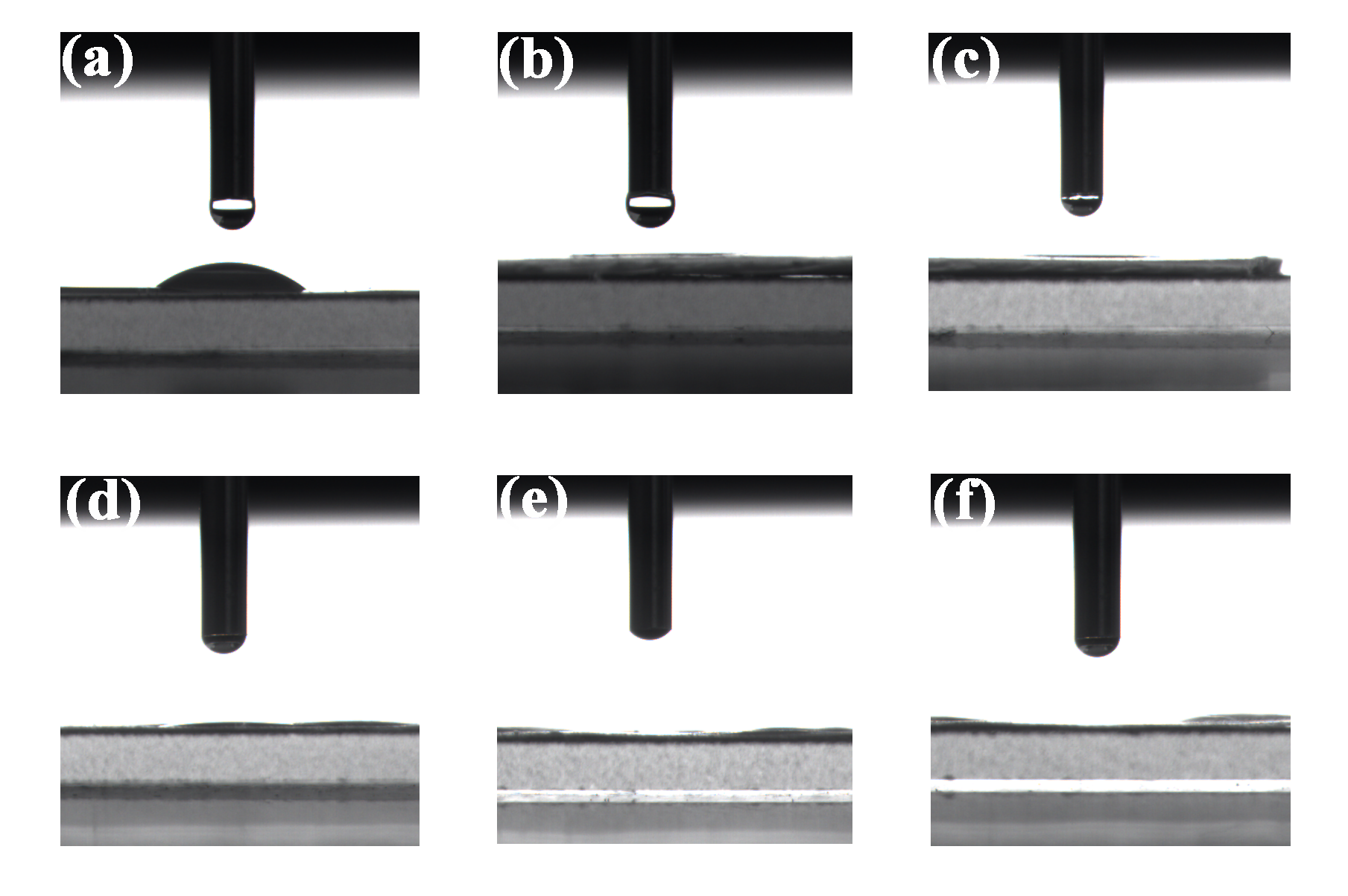


**Fig. S3.** Water contact angle of PAN ultrafiltration membrane prepared by phase inversion (a), PAN nanofibrous membrane (b), cPAN (c), cPAN-Ag0.5 (d), cPAN-Ag1.0 (e) and cPAN-Ag1.5 (f). PAN ultrafiltration membrane was prepared using PAN/DMF/LiCl solution with a mass ratio of 18:80:2.^1^


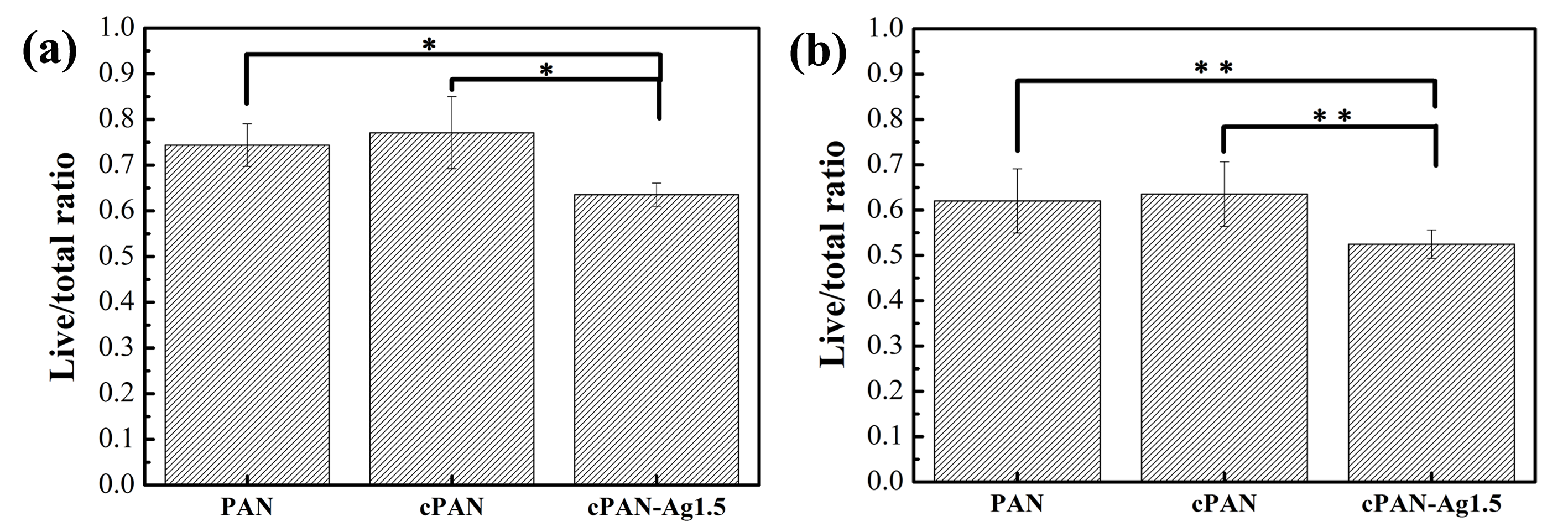


**Fig. S4.** Live/Dead cell ratio of *E. coli* (a) and *B. subtilis* (b) on the surface of different nanofibrous membranes. The symbol “*” and “**”stands for P value < 0.10 and P value < 0.05 respectively.

**References**

1 Qi, *S. et a*l. Influence of the properties of layer-by-layer active layers on forward osmosis performance*. J. Membr. Sc*i**. 423–4**24, 536-542 (2012).
